# Molecular analysis of the chromosomal 16S rRNA gene and *vapA* plasmid gene of Polish field strains of *R*. *equi*

**DOI:** 10.1371/journal.pone.0204024

**Published:** 2018-09-25

**Authors:** Marcin Kalinowski, Zbigniew Grądzki, Łukasz Jarosz, Łukasz Adaszek

**Affiliations:** Department of Epizootiology and Clinic of Infectious Diseases, Faculty of Veterinary Medicine, University of Life Sciences in Lublin, Lublin, Poland; Universidad Nacional Autonoma de Mexico Facultad de Quimica, MEXICO

## Abstract

*Rhodococcus equi* (*R*. *hoagii*) is an opportunistic pathogen commonly found in foals up to 6 months old and animal environment. The *R*. *equi* genome contains genetically stable chromosomal DNA and an 80–90 kb plasmid containing *vapA* gene, responsible for virulence. Most reports from around the world focus on the determination of *R*. *equi* plasmid profiles. Few studies have attempted to determine differences in nucleotide sequences between virulent strains of *R*. *equi* isolated from foals and breeding environment. The aim of the study was to perform a molecular analysis of a fragment of the chromosomal gene encoding the 16S rRNA subunit and the *vapA* plasmid gene of virulent *R*. *equi* strains isolated on Polish studs from foals and from the breeding environment of horses. The sequencing method was used to compare the primary structure of fragments of the chromosomal and plasmid DNA of the virulent *R*. *equi* strains. The sequences of 22 clinical and 18 environmental *R*. *equi* isolates were compared with the sequences of the gene fragments of reference strains available in the NCBI GenBank database. All sequenced 16S rRNA amplicons of Polish field strains were identical and showed 99.5% similarity to the four randomly selected sequences of this gene fragment in the GenBank database. The results confirm that fragments of the 16S rRNA gene of *R*. *equi* strains are highly conserved and do not undergo variation in field conditions. Analysis of the sequencing results for the *vapA* gene fragment of the strains used in our study revealed two polymorphic variants and clear differences between the sequences of strains isolated from foals and from soil samples. Presumably, *R*. *equi* strains present in the breeding environment are more exposed than clinical strains to adverse external factors. This may result in changes in the DNA sequence due to natural selection.

## Introduction

*Rhodococcus equi* also known as *Rhodococcus hoagii* [1] is an opportunistic pathogen commonly found in animal environments. It is the etiological agent of *R*. *equi* pneumonia, a bacterial disease of foals up to 6 months of age [2–6]. *R*. *equi* pneumonia is of considerable economic importance, especially for large studs. Financial losses associated with the disease result mainly from frequent cases of death in foals, among which the mortality rate may be up to 80% if antibiotic treatment is not undertaken. The pathogen is part of the physiological microbiota colonizing the gastrointestinal tract of horses, and sporadically other herbivores, and therefore it is present in the faeces of these animals and contaminates the breeding environment. *R*. *equi* is also well adapted as a soil organism [4–6]. Besides the pathogen is a well-known zoonotic agent causing infections in humans with immune disorders [7–11].

The *R*. *equi* genome, with a total of 5,043,170 bp, contains genetically stable chromosomal DNA and an 80–90 kb virulence plasmid, which is the main determinant of pathogenicity [4,12]. The absence or loss of the plasmid has been shown to result in an inability to multiply *in vitro* in a cell culture of pulmonary macrophages and in the lung tissue of experimentally infected mice and foals [2,13–14]. Sequencing and analysis of plasmid DNA has revealed the existence of 73 open reading frames (ORFs) in four functional regions [4,12,15]. Three of these regions contain genes encoding proteins responsible for the conjugation, replication and stability of the plasmid, as well as proteins that perform other functions that are as yet unknown. The fourth region of the plasmid is a pathogenicity island (PAI), which is 21.3 kb in size and consists of 26 ORFs, whose functions are still largely unknown [15]. On the other hand, the function of 9 ORFs, forming the family of *vap* genes, is known. The family includes 6 genes, *vapA*, *-C*, *-D*, *-E*, *-G* and *-H*, and 3 pseudogenes, *vapF*, -*I* and -*X*. They are responsible for encoding a family of 9 proteins with a molecular weight of 15–22 kDa, i.e. VapA, -C, -D, -E, -F, -G, -H, -I and -X, functionally associated with the virulence of the microbes.

The highly conserved chromosomal gene encoding the 16S ribosomal RNA (rRNA) subunit is of great importance in terms of taxonomy, differentiation of different bacterial species of the genus *Rhodococcus*, and laboratory diagnosis of *R*. *equi* infections. Due to their conserved nature, the nucleotide sequences of this gene are not subject to major modification during phylogenetic development and can therefore be used to detect the genetic material of virulent and avirulent strains of *R*. *equi* [16–19].

In terms of the pathogenesis of *R*. *equi* pneumonia, the most important gene within the plasmid is *vapA*, encoding the VapA protein (Virulence-associated protein A) [13–14,20–21]. All *R*. *equi* strains found to be virulent for mice in laboratory conditions and pathogenic for foals contain the virulence plasmid, and the VapA protein is present in the cell [13–14,22–24]. Moreover, the absence of the plasmid or the VapA protein has been shown to be associated with the loss of virulence, although the presence of the VapA protein alone, without the other proteins of the family, does not enable the bacteria to multiply in murine macrophages or tissues or to colonize the lungs of experimentally infected foals [14,23,25–26]. However, the mechanism of this relationship is not fully understood.

Previous molecular studies conducted around the world on the genetic characteristics of *R*. *equi* have mainly used strains isolated in the USA, South America, Africa and the Far East, i.e. in Japan and Korea [10,27–31]. Among European countries, such studies have mainly been conducted on French strains [3,28,32]. Literature data on the genotypic characteristics of *R*. *equi* strains isolated in Poland are scarce [33–34]. Most reports from around the world focus on the determination of *R*. *equi* plasmid profiles, classifying virulent strains into 12 types: 85-kb types I-IV, 87-kb types I-III and 90-kb types I-V. Few studies have attempted to determine differences in nucleotide sequences between virulent strains of *R*. *equi* isolated from foals and from the breeding environment, which may be useful in research into bacterial variability and the influence of environmental conditions on the virulence of the pathogen.

The aim of the study was to perform a molecular analysis of a fragment of the chromosomal gene encoding the 16S rRNA subunit and the *vapA* plasmid gene of virulent *R*. *equi* strains isolated on Polish studs from foals that died with symptoms of *R*. *equi* pneumonia and from the breeding environment of horses.

## Materials and methods

### Selection and description of studs where the research material was collected

**Fig 1:**
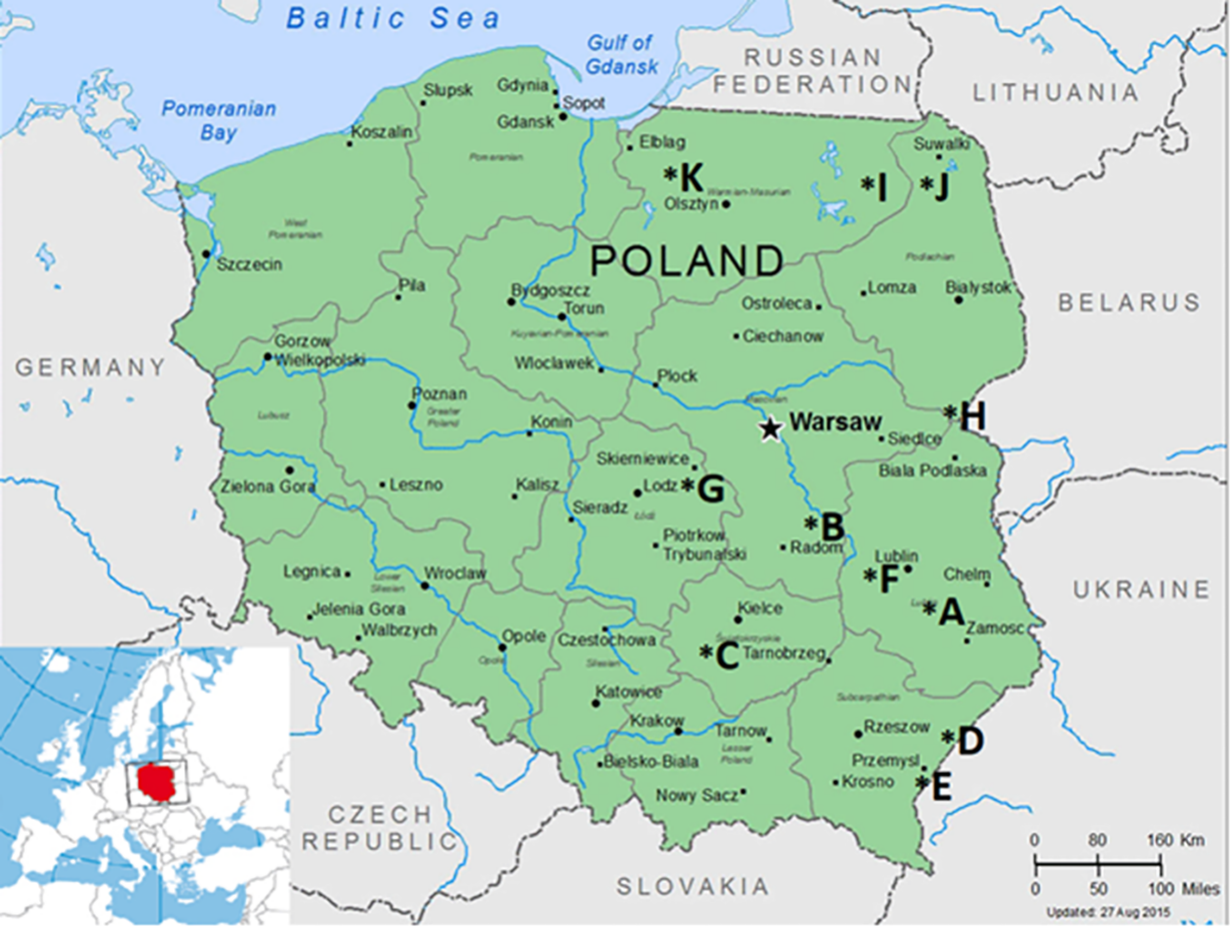
Location of stud farms (A-K) selected for the study.

The studs where the material was collected for testing were selected based on an assessment of their epidemiological state as regards respiratory infections in foals, as well as their veterinary history, including information on suspected and documented cases (confirmed clinically and by additional tests) of pneumonia in foals up to the age of 6 months from which *R*. *equi* was isolated. In total, 11 studs located in central and eastern Poland, designated A to K, were selected for the study (Fig 1).

### Selection of *Rhodococcus equi* strains for laboratory tests

Experimental research was performed on our own collection of *R*. *equi* strains isolated from dead foals in which anatomopathological and laboratory examination confirmed *R*. *equi* pneumonia, as well as strains from the breeding environment, isolated from the top layers of soil collected from studs with enzootic and sporadic *R*. *equi* infection. A total of 40 strains of *R*. *equi* were used in the study, including 22 clinical isolates from 10 studs (A-J), isolated in 2002–2013 from foals that died with symptoms of purulent pneumonia, and 18 strains isolated from soil samples collected in 2009–2016 from 11 studs (A-K).

### Isolation and identification of *R*. *equi* from foals

The clinical strains of *R*. *equi* came from foals that died after being diagnosed with pneumonia. The material for the laboratory tests was collected during the anatomopathological examination from abscesses located in the lung tissue, using a sterile Pasteur pipette. The procedure for isolation and identification of bacteria from pus samples has been described in a previous paper [33]. After identification, clinical strains numbered 1–22 were stored for further testing in a 20% suspension of glycerol in PBS at −70°C.

The study design was reviewed and approved by the Ethics Committee of the University of Life Sciences in Lublin (Poland).

### Isolation and identification of environmental strains of *R*. *equi*

Soil samples were taken from 11 studs located in central and eastern Poland (Fig 1). The top layer of soil, to a depth of about 3 cm, was collected with a teaspoon in a volume of about 10 g and transferred to sterile plastic containers (Medlab, Poland). Five soil samples were collected on each stud from pens, pastures and roads connecting the studs to the pastures, i.e. from the areas frequented by mares with their foals or foals alone. In total, 55 soil samples were collected for laboratory testing. The procedure for isolation and identification of bacteria from soil samples was described in a previous paper [33]. The environmental strains were numbered 23–40 and stored for further testing in a 20% suspension of glycerol in PBS at −70°C.

The field work and collection of soil samples were conducted with the consent of the stud owners. None of the activities undertaken during the field work involved endangered or protected species.

### Species identification and determination of virulence markers of clinical and environmental strains

PCR was used to identify strains isolated from dead foals and from the breeding environment as *R*. *equi* and to determine their virulence, using primers complementary to the sequence of a conserved fragment of the gene encoding the 16S ribosomal RNA (rRNA) subunit and a fragment of the *vapA* plasmid gene according to the methodology described earlier [16,35]. The reference strain *R*. *equi* ATCC 33701, containing the *vapA* gene, was used as a positive control of the PCR reaction.

The sequences of the PCR primers were chosen based on literature data [16,18]. Two primer pairs were used for the reaction. One pair was complementary to the conserved fragment of the 16S ribosomal RNA subunit gene and was used to confirm that the test strains belonged to the species *Rhodococcus equi*. The second pair of primers was complementary to a fragment of the *vapA* plasmid gene encoding the VapA protein responsible for the virulence of the pathogen. Technical details of the PCR reaction were described in a previous paper [18,35].

### DNA sequencing

The sequencing method was used to compare the primary structure of fragments of the chromosomal and extrachromosomal DNA of the *R*. *equi* strains. DNA amplicons, obtained by PCR and initially analysed in an electrophoretic image in a 1.5% agarose gel, were purified on silica columns using the QIAquick PCR Purification Kit (Qiagen, Hilden Germany) according to the manufacturer’s instructions. The purified PCR products were sequenced at the DNA Sequencing and Synthesis Service of the Institute of Biochemistry and Biophysics of the Polish Academy of Sciences in Warsaw. Sequencing results were prepared for analysis using Lasergene MegAlign software (Madison, USA). The sequences of our own *R*. *equi* isolates were compared in order to find any differences between individual strains or with the sequence of the conserved fragment of the gene encoding the 16S rRNA subunit and the *vapA* plasmid gene fragment of reference strains in the NCBI GenBank database, numbered JQ965785.1, NR_041910.1, KF612274.1, AY771327.1 and NC_004854.1, NC_011151.1.

## Results

### Results of PCR

Based on PCR reactions using primers complementary to individual genes, the clinical and environmental strains were identified as belonging to the species *R*. *equi*. All microbial strains had the *vapA* virulence gene. In addition, in previous research [33] to determine the plasmid profiles of clinical and environmental strains of *R*. *equi* occurring in Poland, the majority of strains were classified as presenting the 85-kb type I profile. The exception was strain no. 22, with a profile identified as 87-kb type I [33].

### Results of DNA sequencing

**Fig 2:**
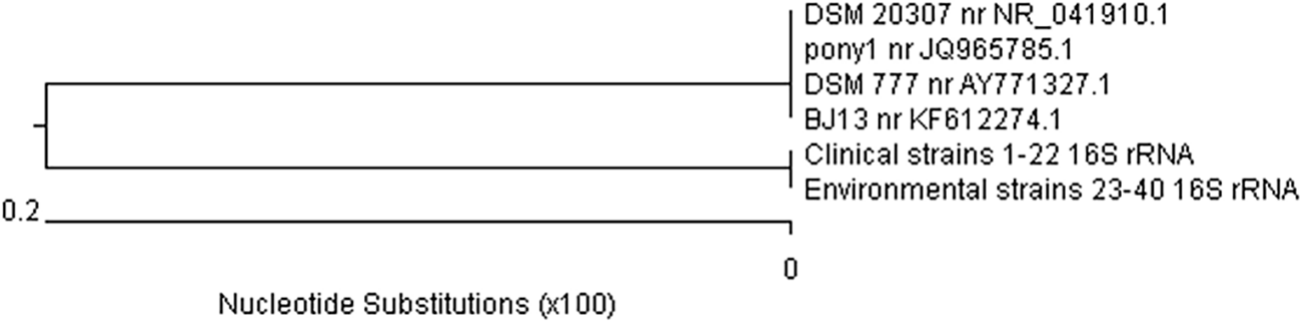
Dendrogram of the similarity of nucleotide sequences of a fragment of the gene (441 bp) encoding the 16S rRNA subunit of *R*. *equi* strains. DSM 20307, pony1, DSM 777, BJ13 –sequences of *R*. *equi* strains from the NCBI GenBank database; 1–22 –clinical strains of *R*. *equi*; 23–40 –environmental strains of *R*. *equi*.

**Fig 3:**
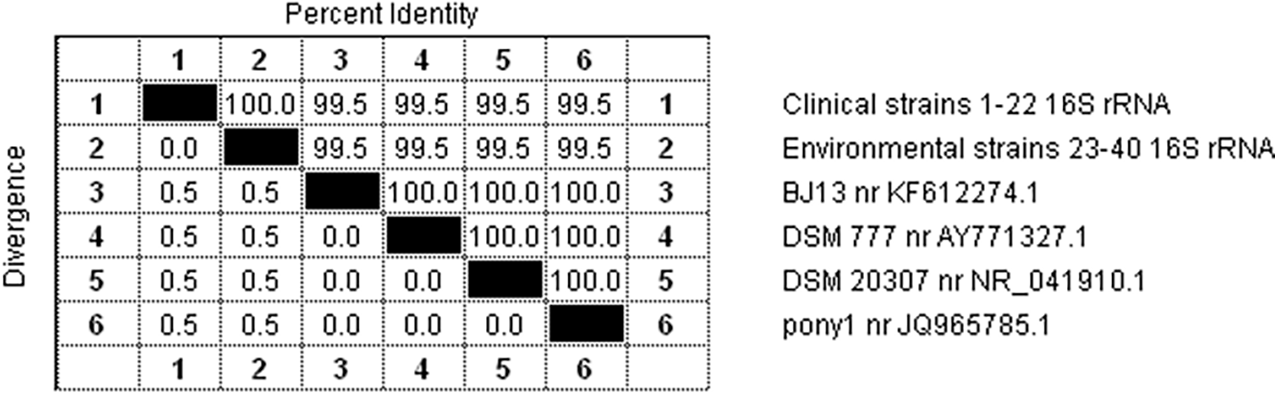
Percentage of identity between sequences of a fragment of the gene encoding the 16S rRNA subunit of *R*. *equi* strains. 1–22 –clinical strains of *R*. *equi*; 23–40 –environmental strains of *R*. *equi*; BJ13, DSM 777, DSM 20307, pony1 –sequences of strains from the NCBI GenBank database.

The products of the two PCR reactions obtained on a template of DNA isolated from each of the 40 Polish field *R*. *equi* strains were sequenced. In the first PCR reaction, a fragment of the conserved gene encoding the 16S ribosomal RNA subunit was amplified. Based on analysis of the nucleotide sequence of the amplicons using Lasergene DNA Star software, a phylogenetic tree was created to illustrate the similarity between DNA sequences of individual *R*. *equi* strains and four sequences of fragments of this gene available in the NCBI GenBank online database, designated DSM 777 no. AY771327.1, DSM 20307 no. NR_041910.1, BJ13 no. KF612274.1 and pony1 no. JQ965785.1 (Fig 2). The data presented in the dendrogram indicate two separate polymorphic variants, the first of which includes all of our own test strains, both clinical and environmental. Among Polish field *R*. *equi* strains, the similarity of the nucleotide sequences of the fragments of the gene encoding the 16S rRNA subunit was shown to be very high, at 100%. The second variant consisted of four randomly selected *R*. *equi* reference strains from France (JQ965785.1), Germany (NR_041910.1), China (KF612274.1) and Taiwan (AY771327.1). The degree of similarity of the nucleotide sequences between our strains and the sequences from the database was high, at 99.5% (Fig 3). Differences were found only in two cases of nucleotide substitution in the sequences of the Polish field strains in comparison to the reference strains. These substitutions were guanine (G) to thymine (T) at position 1 and cytosine (C) to thymine (T) at position 434.

**Fig 4:**
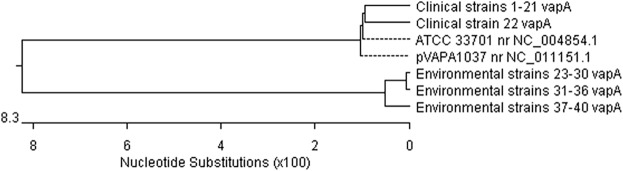
Dendrogram of the similarity of nucleotide sequences of a fragment of the *vapA* gene (875 bp) of *R*. *equi* strains. 1–21 –clinical strains of *R*. *equi* with the 85-kb type I plasmid profile; 22 –clinical strain of *R*. *equi* with the 87-kb type I plasmid profile; ATCC 33701, pVAPA1037 –sequences of *R*. *equi* reference strains from the NCBI GenBank database; 23–30, 31–36, 37–40 –environmental strains of *R*. *equi*.

**Fig 5:**
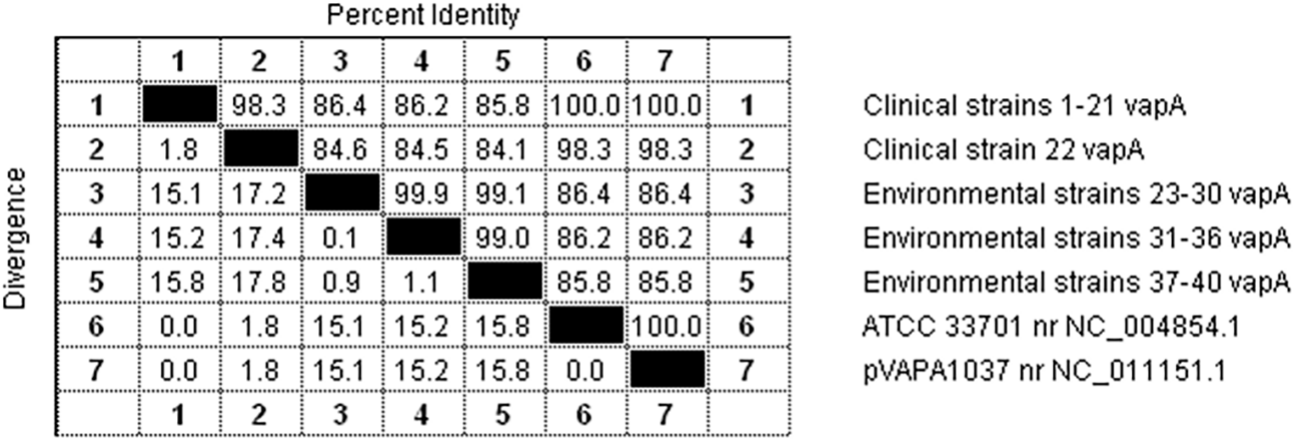
Percentage of identity between sequences of a fragment of the *vapA* gene of *R*. *equi* strains. 1–21 –clinical strains of *R*. *equi* with the 85-kb type I plasmid profile; 22 –clinical strain of *R*. *equi* with the 87-kb type I plasmid profile; 23–30, 31–36, 37–40 –environmental strains of *R*. *equi*; ATCC 33701, pVAPA1037 –sequences of *R*. *equi* reference strains from the NCBI GenBank database.

The second PCR reaction amplified a fragment of the *vapA* plasmid gene encoding the protein of the same name, responsible for the virulence of the pathogen. Analysis of the nucleotide sequences of this gene using computer software made it possible to create a dendrogram illustrating the similarity between the sequences of the Polish field *R*. *equi* strains and the sequences of two reference strains available in the NCBI GenBank database: ATCC 33701 no. NC_004854.1 from Japan and pVAPA1037 no. NC_011151.1 from the UK (Fig 4). Analysis of the phylogenetic tree created by comparing fragments of the *vapA* gene sequence indicates, as in the case of the gene encoding the 16S rRNA subunit, two distinct polymorphic variants. In these variants, a clear separation can be seen between clinical strains from dead foals and environmental strains isolated from soil samples. The first variant included sequences from all clinical strains from our own collection (1–22) and both sequences of the reference strains. The similarity of the nucleotide sequences between nearly all isolates within the first variant was very high, at 100%. The exception was the sequence from strain no. 22, which had a different plasmid profile (87-kb type I) from that of the other strains. Although the sequence derived from this strain was classified as the same polymorphic variant as the sequences of the other clinical and reference strains, the degree of similarity between them was lower, at 98.3% (Fig 5). The second polymorphic variant consisted of the sequences of the environmental *R*. *equi* strains (23–40), which had a high degree of similarity ranging from 99.0% to 99.9%. The similarity between the environmental strains and the clinical and reference strains in the first polymorphic variant ranged from 85.8% to 86.4%. Slightly lower similarity, ranging from 84.1% to 84.6%, was demonstrated between the sequence of strain 22, isolated from a dead foal, and the environmental strains (Fig 5).

## Discussion

Our analysis of the sequences of both genes is the first attempt at a molecular characterization of Polish field *R*. *equi* strains. The products obtained by amplifying a fragment of the conserved gene encoding the 16S ribosomal RNA subunit (rRNA) and a fragment of the *vapA* plasmid gene were sequenced. Fragments of both genes are currently widely used in microbiological diagnostics and molecular studies of infections caused by *R*. *equi* to distinguish virulent and avirulent strains and to confirm a diagnosis of *R*. *equi* infection [16–18,35–36]. Learning the nucleotide sequence of both genes provides important information that can be used in molecular analysis of *R*. *equi* strains by determining the degree of differentiation of isolates derived both from clinical material and from the breeding environment. The nucleotide sequences of a fragment of the chromosomal gene encoding the 16S rRNA subunit are highly conserved and do not undergo major modifications in the phylogenetic development of the bacteria [16,19]. However, the variable regions occurring within these sequences are characteristic for individual strains, which makes it possible to identify and differentiate them [16]. Currently, the rRNA gene sequences are known for most microorganisms, including *R*. *equi* [18,37]. Amplification of this gene for diagnostic purposes enables the detection of all *R*. *equi* strains, including avirulent ones isolated mainly from the breeding environment and virulent ones isolated mainly from clinical material, but also from soil samples. All sequenced 16S rRNA amplicons obtained from our own strains, irrespective of their source, were identical and showed 99.5% similarity to the four randomly selected sequences of this gene fragment in the GenBank online database. Interpretation of the minor differences shown in our study in the nucleotide sequence of the 16S rRNA gene fragment is not straightforward. In addition to the natural variation in sequences of strains of the same species, the differences identified may also be the result of different sequencing techniques. This possibility should be taken into account, given that the differences shown pertain to our own clinical and environmental strains and those collected in the NCBI GenBank online database. Therefore we cannot rule out differences in the methodology used to determine the nucleotide sequence of the two groups of strains. Differences of only two bases can also be the result of sequencing errors or SNPs (Single Nucleotide Polymorphism) within gene duplicates in one strain. The results confirm that, as in the case of other bacteria, fragments of the gene encoding the 16S rRNA subunit of *R*. *equi* strains are highly conserved and do not undergo variation in field conditions on studs. Similar results have been published by Bell et al. [16], who pointed out that the analysis of nucleotide sequences within the 16S RNA gene enables not only precise identification of *R*. *equi*, but also differentiation of the pathogen from closely related species of the genera *Nocardia*, *Gordonia*, *Dietzia*, *Tsukamurella*, *Corynebacterium* and *Mycobacterium*. The conserved nature of the 16S rRNA gene has also been confirmed in studies by Sellon et al. [19] and by Coenye and Vandamme [38].

In our study on the genotypic characteristics of Polish field *R*. *equi* strains, we also sequenced a fragment of the *vapA* plasmid gene coding for the VapA surface protein, important in terms of the virulence of the pathogen. Analysis of the sequencing results for the gene fragment of the strains used in our study revealed two polymorphic variants and clear differences between the sequences of strains isolated from foals and from soil samples. All sequences of clinical strains, except for no. 22, showed 100% similarity among one another and with the sequences of the Japanese and British strains from the GenBank database. The sequence similarity of strain 22 to the other strains was high, at 98.3%, but there were minor differences between the sequences of strains belonging to the two groups. The results obtained by sequencing are consistent with the earlier assessment of the plasmid profiles of our own strains isolated from dead foals, which showed that strain no. 22 also had a different virulence plasmid profile from the other strains. Data published by Makrai et al. [39] indicate that strains with this plasmid profile can be found in Europe, including France, Italy, Turkey, and Germany, but also in both Americas and Australia. The single case of isolation of a strain with this profile in Poland should be considered an accidental introduction, whose possible causes have been discussed in a previous paper [33]. In view of the common occurrence of the 85-kb type I profile around the world, and to better understand the structure and function of the virulence plasmid and *vapA* gene, Takai et al. [40] determined the complete plasmid sequence of two reference strains, ATCC 33701 and 103S. The results showed complete similarity between the two reference strains, which confirms the results of our own comparison of the sequences of *vapA* gene fragments of Polish field *R*. *equi* strains. Duquesne et al. [3], in a comparative study, analysed the complete sequence of the plasmid presenting the second most common profile, i.e. 87-kb type I. Although this plasmid is 2490 base pairs longer than the plasmid with profile 85-kb type I, their sequences proved to be highly conserved, and the similarity between them was 95.8%. This finding also confirms the results of our own study, in which the sequence similarity of the *vapA* gene fragments from both groups was even higher, at 98.3%. Slightly different results were obtained in the comparative analysis of the nucleotide sequences of domestic environmental and clinical strains presenting the same virulence plasmid profile, i.e. 85-kb type I. The sequence similarity between these strains and in comparison with the reference strains was lower, ranging from 85.8% to 86.4%, although the similarity of the environmental strains among one another was high, ranging from 99.0% to 99.9%. The reasons for these discrepancies can be traced to the origin of the samples from which the bacteria were isolated. Presumably, *R*. *equi* strains present in the breeding environment are more exposed than clinical strains to adverse external factors. This hypothesis is supported by the analysis of the biochemical properties of environmental and clinical *R*. *equi* isolates, which indicates that the environmental strains produced a larger and more diversified spectrum of enzymes than the clinical isolates [10,12]. Such conditions may result in a change in the DNA sequence due to the natural selection process. The differences described may also be linked to mutations occurring within the plasmid DNA of bacteria in the external environment. Some authors also suggest that after entering the environment, virulent strains of *R*. *equi* may lose the virulence plasmid, becoming avirulent, and then regain the plasmid from other virulent strains via co-option [2,4,12,15]. This phenomenon may cause minor differences in the nucleotide sequence of the *vapA* gene, resulting in new variants of virulent strains. It is worth noting that the differences in the nucleotide sequences analysed in the present study for the strains of both polymorphic variants were not associated with a change in the plasmid profile, and despite the changes observed in the sequences, the environmental and clinical strains presented the same profile, 85-kb type I.

The DNA sequencing method made it possible to obtain more precise information on the structure of the *R*. *equi* virulence plasmid. Letek et al. (107) analysed the nucleotide sequences of the plasmid of the *R*. *equi* reference strain pVAPA1037 and showed that it consisted of four regions. Three regions are genetically stable and have highly conserved sequences, constituting a standard for most virulent strains of *R*. *equi* isolated from horses. The fourth region is a variable region containing a pathogenicity island (PAI). In strains isolated from horses, this island contains 9 *vap* genes, including *vapA*, which is important for the virulence of the bacterium. The research on the *R*. *equi* genome conducted by the authors cited showed that the pathogenicity island containing the *vapA* gene is highly susceptible to nucleotide sequence changes as a result of duplication, translocation and inversion of genes as well as insertions and deletions of nucleotides that can lead to the emergence of new virulent strains of the pathogen. In addition, the authors suggest that changes in the sequences of the *vap* family genes, including *vapA*, do not necessarily affect the type of plasmid profile, as the sequence of this gene is only a small part of the sequence of the entire pathogenicity island. This hypothesis is confirmed by the results of our own study, which demonstrated the existence of one plasmid profile in the clinical and environmental strains, irrespective of differences in the nucleotide sequence of the *vapA* gene. On the other hand, changes at the genome level, translating into a change in the amino acid sequence of VapA proteins, may determine the adaptation of *R*. *equi* to other animal species and humans. This is evidenced by the latest reports by Willingham-Lane et al. (213), indicating the ability of *R*. *equi* strains having a plasmid with the *vapA* gene to survive and multiply inside macrophages of horses, pigs and mice. A similar view is presented by Duquesne et al. (46), who believe that changes in nucleotide sequences within the *vapA* gene may in the future lead to new variants of plasmids determining greater virulence of strains and increased potential to adapt to species other than horses. Further research should therefore be conducted on the molecular characteristics of *R*. *equi* strains, especially focused on the sequence analysis of the entire pathogenicity island (PAI) responsible for the virulence of *R*. *equi* strains.
